# Self-reported indications for antidepressant use in a population-based cohort of middle-aged and elderly

**DOI:** 10.1007/s11096-016-0371-9

**Published:** 2016-09-01

**Authors:** Nikkie Aarts, Raymond Noordam, Albert Hofman, Henning Tiemeier, Bruno H. Stricker, Loes E. Visser

**Affiliations:** 1 Department of Epidemiology, Erasmus MC – University Medical Center Rotterdam, Rotterdam, The Netherlands; 2 Department of Internal Medicine, Erasmus MC – University Medical Center Rotterdam, Rotterdam, The Netherlands; 3 Department of Psychiatry, Erasmus MC – University Medical Center Rotterdam, Rotterdam, The Netherlands; 4 Department of Child and Adolescent Psychiatry, Erasmus MC – University Medical Center Rotterdam, Rotterdam, The Netherlands; 5 Inspectorate of Health Care, Utrecht, The Netherlands; 6 Apotheek Haagse Ziekenhuizen – HAGA, The Hague, The Netherlands

**Keywords:** Antidepressive agents, Depression, Off-label use, Self-report, The Netherlands

## Abstract

*Background* Population-based studies investigating indications for antidepressant prescribing mostly rely on diagnoses from general practitioners. However, diagnostic codes might be incomplete and drugs may be prescribed ‘off-label’ for indications not investigated in clinical trials. *Objective* We aimed to study indications for antidepressant use based on self-report. Also, we studied the presence of depressive symptoms associated with the self-reported indications. *Setting* Our study population of antidepressant users was selected based on interview data between 1997 and 2013 from the prospective population-based Rotterdam Study cohort (age >45 years). *Method* Antidepressant use, self-reported indication for use, and presence of depressive symptoms (Center for Epidemiological Studies Depression Scale) were based on interview. Self-reported indications were categorized by the researchers into officially approved, clinically-accepted and commonly mentioned off-label indications. *Main outcome measures* A score of 16 and higher on the Center for Epidemiological Studies Depression Scale was considered as indicator for clinically-relevant depressive symptoms. *Results* The majority of 914 antidepressant users reported ‘depression’ (52.4 %) as indication for treatment. Furthermore, anxiety, stress and sleep disorders were reported in selective serotonin reuptake inhibitor and other antidepressant users (ranging from 5.9 to 13.3 %). The indication ‘pain’ was commonly mentioned by tricyclic antidepressant users (19.0 %). Indications were statistically significantly associated with higher depressive symptom scores when compared to non-users (n = 10,979). *Conclusions* Depression was the main indication for antidepressant treatment. However, our findings suggest that antidepressants are also used for off-label indications, subthreshold disorders and complex situations, which were all associated with clinically-relevant depressive symptoms in the middle-aged and elderly population.

## Impacts on practice

- Antidepressant use should not be used as a marker for subclinical depressive symptoms in pharmacoepidemiological research.
- Antidepressants are prescribed for subthreshold psychiatric symptoms, complex, and possible off-label, indications.
- Off-label antidepressant use seems common in the elderly population and appropriateness of prescribing should be further investigated.

## Introduction

Antidepressant use has increased substantially in the last decades [1–3]. It has been suggested that antidepressants are prescribed too easily [4, 5], or that antidepressants are prescribed for a wider range of indications [2, 6]. Antidepressants are not only prescribed for depressive disorders, but also for other approved (i.e. anxiety) and unapproved indications, referred to as (clinically-accepted) off-label indications, such as sleep disorders and neuropathic pain [7]. A broadening in the number of indications for antidepressant use might explain the rise in antidepressant use over time [2, 6]. Pharmacoepidemiological studies that investigated indications for antidepressant use often registered indications based on clinical diagnostic codes from medical records [2, 7–10] or were based on diagnoses from structured interviews with general practitioners (GPs) [11, 12]. However, diagnostic codes might be incomplete if not all indications were under investigation, subthreshold psychiatric symptoms were not registered or because of diagnostic uncertainty by the GP [2, 7–9].

Studies which investigate reasons for antidepressant prescribing from a patients’ perspective give insight into the original symptoms and primary condition as experienced by the patient. Besides the traditional indications, subthreshold disorders, physical comorbidities and life events have been associated with use of antidepressants [6, 13–15]. Further characterization of these individuals and their, non-psychiatric, indications is needed.

## Aim of the study

Our aim was to investigate indications for antidepressant treatment based on patients’ self-reports in a population-based cohort of middle-aged and elderly. Additionally, we aimed to assess presence of depressive symptoms, as we hypothesize that all indications might be accompanied by comorbid depressive symptoms.

## Ethics approval

The Rotterdam Study has been approved by the Medical Ethics Committee of the Erasmus MC and by the Ministry of Health, Welfare and Sport of the Netherlands, implementing the “Wet Bevolkingsonderzoek: ERGO (Population Studies Act: Rotterdam Study)”. All participants provided informed consent to participate in the study.

## Method

### Study setting

The study was conducted within the prospective population-based Rotterdam Study. The Rotterdam Study was initiated in 1990, and investigates the incidence of, and risk factors for, several age-related diseases. Participants are continuously followed for morbidity and mortality, and follow-up visits are conducted every 4–5 years with an extensive set of examinations (e.g. ECG, MRI-scan, questionnaires). After extension over the years, the study comprises a total of 14,926 participants. All participants were aged 45 years or older at baseline. Detailed information on design, objectives and methods of the Rotterdam Study has been published elsewhere [16, 17].

### Study population

From 1997 onwards, participants were interviewed every 4–5 years by research assistants about their current drug use and indication for use (N = 11,860). We included participants who reported antidepressant use at one of the interview rounds (between 1997 and 2013). Home interviews of other participants, with complete interview data, were included as reference population of non-users.

### Home interviews

Participants were asked to present all their drug containers during the home interview. Drug names, dosages, and regimen were registered by research assistants. Antidepressants were selected, based on their Anatomical Therapeutic Chemical (ATC) code (ATC code = N06A), and categorized into tricyclic antidepressants (TCAs, ATC-code = N06AA), selective serotonin reuptake inhibitors (SSRIs, ATC-code = N06AB) and other antidepressants (ATC-code = N06AF/AG/AX). St John’s wort was not taken into account. Moreover, participants were asked for which indications the specific drugs were taken. The symptoms or disorders mentioned by the participant were registered as ‘free text’, without interpretation or adjustment by the research assistants. Two researchers (NA and RN) independently categorized the complete list of symptoms and disorders into eight groups. Discrepancies were discussed to reach final consensus. The categories would represent disorders and related symptoms, and were based on officially approved and clinically-accepted indications extended with common off-label indications mentioned in previous literature (Table 1) [7, 9, 18].

**Table 1:** Overview of indications and their corresponding self-reported symptoms, and the registered approved or clinically-accepted antidepressants

Category	Reported symptoms	Approved antidepressants	Clinically-accepted antidepressants ^a^
Depression	Depression, anti-depressant, feeling down, discouraged	All	
Anxiety	Anxiety, panic, hyperventilation	Clomipramine, venlafaxine, duloxetine and SSRIs^b^	Imipramine
Stress	Stress, burn-out, restlessness, soothing		
Sleep disorders	Insomnia, to fall asleep, sleep		
Headache/migraine	Headache, migraine		
Pain	Pain, nerve pain, hernia, fibromyalgia, shingles	Duloxetine	Amitriptyline
Other indications	Parkinson’s disease, menopause, tingling legs, general mental problems		
Unknown	No answer, unknown		

*SSRIs* selective serotonin reuptake inhibitors

^a^Clinically-accepted antidepressants are mentioned in the national therapeutic monitoring system

^b^Some SSRIs are only registered for specific anxiety disorders, however we consider all SSRIs registered as we cannot distinguish these specific anxiety disorders

As part of the home interview, presence of depressive symptoms in the past week before interview were screened for with a Dutch version of the Center for Epidemiological Studies Depression Scale (CES-D) [19]. A score of 16 and higher was considered as an indicator for clinically-relevant depressive symptoms [20].

### Statistical analyses

We included the first eligible interview of participants. Self-reported indications were presented for the total group of antidepressant users and stratified by antidepressant class (TCA, SSRI, other). Participants could report multiple antidepressants and indications in an interview. In a subsample analysis, we excluded participants without information regarding cognitive functioning or with possible cognitive impairment (Mini-Mental State Examination score ≤23 [21]), as cognitive impairment may affect the validity of self-reported data. Also, indications for treatment were stratified by age (≤65, >65 years) and sex. In an additional analysis, we included follow-up interview rounds to map whether participants reported antidepressant use at multiple rounds and whether indications for treatment were consistent over the rounds.

The median depression score and the percentage of participants with clinically relevant depressive symptoms were presented for all indications of treatment and for non-users. Median scores and percentages for every indication category were compared to non-users or compared to the group with indication ‘depression’ with a Mann–Whitney U, or Pearson Chi square test. For these analyses regarding the depression score, we categorized participants who reported multiple antidepressants and indications in an interview as a separate category. A *p* value below 0.05 was considered statistically significant and IBM SPSS Statistics (version 21.0, IBM Corp., Somers, NY, USA) was used for analyses.

## Results

Of the 11,860 participants, a total of 914 (7.7 %) individuals reported to be current antidepressant user at one or multiple interview rounds. At the first eligible interview round, the mean age of the antidepressant users was 67.3 years (SD 10.7), 72.3 % were women, and most participants were prescribed an SSRI (54.5 %).

Depression was most commonly reported as indication for treatment in the antidepressant users (52.4 %, Table 2). SSRIs and other antidepressants were most often used for depression, anxiety and stress symptoms, although other antidepressants were also prescribed for sleep disorders and other indications. Of the TCA users, only 29.6 % reported depression as an indication, while stress (14.4 %), sleep disorders (10.4 %) and pain (18.9 %) were also frequently reported. In total, antidepressant use was in 60.1 % of the reported indications for approved indications, 5.1 % for clinically-accepted indications (i.e. mostly amitriptyline for pain) and 36.6 % would be considered off-label. Other reported indications were menopausal complaints, tingling or restless legs, Parkinson’s disease and general mental health problems (Table 1). Percentages did not materially change when we excluded participants with possible cognitive impairment (subsample n = 717, results not shown). Moreover, stratification by age (cut-off ≤65, >65 years) showed higher percentages of depression (61.0 vs. 45.0 %) and anxiety-related (14.4 vs. 3.9 %) indications in the younger population than in the older population, while the older population had a higher percentage of stress related (9.0 vs. 16.5 %) and unknown indications (5.0 vs. 20.8 %). Stratification by gender showed comparable indications for treatment for men and women (results not shown). A total of 358 participants reported antidepressant use at two measurement rounds. Self-reported indications for depression and anxiety remained relatively stable over time with 78.4 % reporting depression or anxiety at both interview rounds. In participants who initially reported other or unknown indications, 40.7 % did report depression or anxiety at follow-up measurement round.

**Table 2:** Self-reported indications for all antidepressant users and stratified by type of antidepressant

	Total	TCA	SSRI	Other
	N = 914	N = 270	N = 498	N = 153
	N (%)	N (%)	N (%)	N (%)
Reported indication for use^a^				
Depression	479 (52.4)	80 (29.6)	307 (61.6)	92 (60.1)
Anxiety	80 (8.8)	6 (2.2)	65 (13.1)	9 (5.9)
Stress	119 (13.0)	39 (14.4)	66 (13.3)	14 (9.2)
Sleep disorders	45 (4.9)	28 (10.4)	6 (1.2)	11 (7.2)
Headache/migraine	10 (1.1)	9 (3.3)	1 (0.2)	0 (0.0)
Pain	54 (5.9)	51 (18.9)	1 (0.2)	2 (1.3)
Other	48 (5.3)	19 (7.0)	18 (3.6)	11 (7.2)
Unknown	123 (13.5)	46 (17.0)	57 (11.4)	20 (13.1)

Number of separate antidepressants and indications do not add up to the total number of unique participants (n = 914), as participants could report multiple antidepressants and indications at one interview round

*TCA* tricyclic antidepressants, *SSRIs* selective serotonin reuptake inhibitors

^a^Reported indications for antidepressant use represent disorders and related symptoms. See Table 1

All indications, except headache, were associated with a significantly higher percentage of participants with clinically-relevant depressive symptoms or a higher median CES-D score than the non-users (8.1 %, median 2.0, interquartile range 0.0–7.0). Of the antidepressant users with the self-reported indication depression, 36.9 % also had clinically-relevant depressive symptoms as measured by questionnaire. Nonetheless, of the users who exclusively reported another indication for use, 23.4 % had clinically-relevant depressive symptoms ranging from 15.9 to 29.2 % over the different indication categories (Table 3). However, the percentage with clinically-depressive symptoms was significantly lower in participants who exclusively reported another indication for use, when compared to the indication ‘depression’. An exceptional category was the group with multiple indications or antidepressants at an interview round. These participants had a median depression score of 12.5 (interquartile range 4.5–20.0), with a total of 42.9 % of participants with clinically-relevant depressive symptoms, which was significantly higher when compared to the non-users group, but similar to the users with the self-reported indication depression (Table 3).

**Table 3:** Self-reported indications for antidepressant use and presence of depressive symptoms

	N	Median CES-D score Median (IQR)	Presence of depressive symptoms^d^ N (%)
Non–use	10,797	2.0 (0.0–7.0)	877 (8.1)
*Reported indication for use* ^a^			
Depression	436	11.0 (3.0–20.8)^b^	161 (36.9)^b^
*Single indications except depression*	410	8.0 (3.0–15.0)^b,c^	96 (23.4)^b,c^
Anxiety	59	5.0 (2.0–13.0)^b,c^	10 (16.9)^b,c^
Stress	105	9.5 (3.0–16.5)^b^	27 (24.7)^b,c^
Sleep disorders	33	7.0 (3.0–15.5)^b^	8 (24.2)^b^
Headache/migraine	8	1.0 (0.0–14.3)	2 (25.0)
Pain	48	5.0 (1.0–13.8)^b,c^	9 (18.8)^b,c^
Other	44	6.5 (2.3–13.0)^b,c^	7 (15.9)^c^
Unknown	113	9.0 (4.5–16.9)^b^	33 (29.2)^b^
Multiple indications	42	12.5 (4.5–20.0)^b^	18 (42.9)^b^

Complete caseset analysis. St John’s wort was not taken into account as antidepressant and excluded from the non-use group. N does not correspond with total N in Table 2: CES-D data was missing for 26 antidepressant users, and participants with multiple indications or antidepressants were categorized in a separate group (‘multiple’), and were only taken into account once

*CES-D*Center for Epidemiological Studies Depression Score, *IQR* interquartile range

^a^Reported indications for antidepressant use represent disorders and related symptoms. See Table 1

^b^Significantly different from non-use (*p* < 0.05)

^c^Significantly different from the indication ‘depression’ (*p* < 0.05)

^d^Presence of depressive symptoms is based on a Center for Epidemiological Studies Depression Score of 16 or higher

## Discussion

In line with other studies, we observed in our study population of older adults that depression was the most frequently reported indication for antidepressant use [2, 7, 10, 11]. Of those who reported depression as indication for treatment, around 40 % actually reported clinically-relevant depressive symptoms based on assessment with the CES-D. Possibly, antidepressants were prescribed for mild depressive symptoms, or participants were on maintenance treatment after successful antidepressant treatment [13, 22].

As hypothesized, more than 20 % of users also reported clinically-relevant depressive symptoms for indications other than depression, and had a higher depression score when compared to non-users. Two possible explanations will be discussed. First, we were able to capture mild or isolated symptoms, such as restlessness and fatigue, which do not conform to the diagnostic label depression according to the DSM-IV criteria. Yet, these are symptoms which could be part of a possible depression. Second, symptoms such as depression, social problems, distress and physical symptoms are highly correlated and often occur concomitantly. Depression might be a secondary indication next to the reported primary condition [11, 23–25]. 40 % of the participants who used antidepressants during two interviews reported anxiety or depression at follow-up, while they reported another indication at baseline. The statement that the profile of antidepressant use is complex is further supported by the fact that participants with multiple indications also reported a high level of depressive symptoms, even similar to the group who reported depression as indication for treatment. Nevertheless, antidepressant use cannot be used as a marker for subclinical depressive symptoms. In most users which exclusively reported another indication, the overall percentage of depressive symptoms was significantly lower when compared to those who reported depression as indication for treatment.

In contrast, almost 80 % of users with an indication other than depression did not report concurrent depressive symptoms. In this case, the reported (off-label) indications such as anxiety, pain, stress or sleeping disorders were the main reason for prescribing according to the patient. For example, we observed that almost one fifth of all TCA prescriptions were reported as being used for pain. This proportion was higher than in previous studies [7, 10], which might be due to the high average age of our population because pain is highly prevalent in the elderly [26]. The off-label indication ‘stress’ was also frequently mentioned in our study. This might relate to participants who experience psychosocial problems and distress, without clinical depressive symptoms which justified antidepressant prescribing. Previous studies already reported that, besides the traditional indications, combinations of other mental complaints, multi-morbidity, psychosocial problems and social distress are also important reasons to prescribe antidepressants [5, 6, 13–15]. This may be particularly important in our elderly population as elderly experience more life events, physical impairment and general health complaints. This is confirmed after our stratification on age as the percentage with stress related indications is even higher in the elderly population. Although prescriptions for these indications seem off-label, no conclusions can be drawn regarding the appropriateness of the antidepressant prescribing in our study. Information from detailed individual medical records would be required.

Previous studies already reported that antidepressants are being prescribed for other, possible off-label, indications, however these results are most often based on clinical diagnostic codes [2, 7–10], which does not provide a complete representation [2, 7–9]. Other more qualitative studies provide a more complete representation of the complex and different combinations of indications, but they were always influenced by a physician’s judgement or still focused on psychiatric disorders. We provide a unique insight from a patients’ perspective into the original symptoms and primary condition as experienced by the patient. We report a complete overview of all possible indications with antidepressant use in a daily practice setting.

Other strengths of our study are the population-based setting and the availability of interview data from participants on antidepressant use, indication for use and depression score at the same point in time. However, some limitations should be addressed. First, in a prospective cohort study such as the Rotterdam Study a healthy volunteer bias may be considered as a sort of selection bias [27]. Second, indications based on self-report might be biased as they relate to individual cognitive and linguistic abilities. However, results did not materially differ after exclusion of participants with possible mild cognitive impairment. Third, there may be a discrepancy between a physicians intended indication for use and the patients perception on it. Unfortunately, we did not have the physicians diagnoses to compare with the self-reported indication for use. Fourth, reported symptoms were manually categorized by two independent researchers in different indication groups. These categories might not be mutually exclusive from each other as symptoms often co-exist and are not always distinctive for one specific indication. Fifth, the majority of our analyses were based on cross-sectional data and do not capture changes in symptoms over time.

## Conclusion

Our results suggest that apart from the product-labelled indications, antidepressant use is common for self-reported off-label indications, subthreshold disorders and distress in the middle-aged and elderly population. Clinically-relevant depressive symptoms were observed for indications other than depression, which suggests that there is a high correlation between physical problems, psychological distress and depression. Thus, according to antidepressants users they not only use antidepressants to treat the traditional indications of depression and anxiety. Further research is needed to study whether antidepressant use is justified in these cases. The balance between possible effectiveness and risk of adverse drug reactions should be weighted carefully, especially in the elderly population which are at high risk of adverse drug reactions and drug-drug interactions.
